# Examining Narcissistic Traits in Relation To Reactive and Proactive Aggression in Children At-Risk for Attention Deficit/Hyperactivity Disorder

**DOI:** 10.1007/s10578-025-01824-4

**Published:** 2025-04-10

**Authors:** Cathleen Odar Stough, Veronica Mehl, Stephen P. Becker, Leanne Tamm

**Affiliations:** 1 Department of Psychology, University of Cincinnati, College of Arts and Sciences, Cincinnati, OH USA; 2 Cincinnati Children’s Hospital Medical Center, Division of Behavioral Medicine and Clinical Psychology, Department of Pediatrics, University of Cincinnati College of Medicine, Cincinnati, OH USA

**Keywords:** Narcissism, Proactive aggression, Reactive aggression, ADHD, Psychopathy

## Abstract

The current study examined narcissistic traits in relation to proactive and reactive functions of aggression in a sample of children at-risk for attention-deficit/hyperactivity disorder (ADHD). Children ages 7–13 years (*N* = 110) were recruited from a pediatric ADHD assessment clinic. Caregivers completed the Antisocial Process Screening Device. Caregivers and teachers completed a measure of proactive and reactive aggression. In regression analyses controlling for ADHD and oppositional defiant disorder symptom severity and sex, narcissism was uniquely associated with higher caregiver-reported proactive aggression but not reactive aggression. Findings suggest that narcissism is uniquely related to proactive aggression in children at-risk for ADHD. Consideration of narcissistic traits may aid the prediction of behavioral issues and aggression in children and may be a potential intervention target. Future research should investigate if clinical interventions addressing narcissistic behaviors are effective for reducing proactive aggression in children.

Aggressive behavior is common among children with attention-deficit/hyperactivity disorder (ADHD; [65, 69]), and a large proportion of youth with ADHD display clinically significant aggression [58]. Deficiencies in inhibitory control mechanisms and social information processing have been implicated in increased risk of aggressive behaviors among children with ADHD [9, 35]. This risk for aggression is concerning given children with elevated and persistent patterns of aggressive behavior are at greater risk for negative outcomes over time (e.g., poor employment, education, general and mental health; [7]). Further, co-occurring aggression can worsen difficulties that children with ADHD face, including greater likelihood of persistent ADHD symptoms and serious lifelong impairments in functioning [11, 58]. Aggression can be classified as having two functions: proactive and reactive; proactive aggression is unprovoked, intentional, and has an instrumental function wherein it is used for the purpose of personal gain or to dominate or coerce others [15, 38, 52]. In contrast, reactive aggression arises in response to a perceived threat and serves a defensive function [15, 60]. Although highly correlated, proactive and reactive aggression identify youth with different patterns and outcomes [17]. Given the risk for poor outcomes among children with ADHD if aggressive behaviors persist, identifying predictors of functions of aggression is critical.

Narcissism, including sub-pathological narcissism, has been related consistently to both reactive and proactive aggression, especially in situations where aggression has been provoked [36]. However, much of the research demonstrating this association has been completed in laboratory settings and with college students, and there has been a call for investigation of the relationship in other samples, including children [36]. Researchers have noted that narcissism is a relatively neglected but important predictor of childhood conduct problems [2]. General population studies in children suggest that the presence of narcissistic traits increases the risk of persistent antisocial behavior [4, 33, 42, 48]. Youth with narcissistic traits may be at-risk for engaging in greater aggressive behaviors due to less sensitivity to punishment cues, threat and distress cues, and deficits in moral reasoning and empathy [10]. Understanding aggression and narcissistic traits among youth with ADHD is of particular interest because they present with high rates of both proactive and reactive aggression, conduct problems, and psychopathic traits, including narcissism [6, 43, 54, 59]. Children with ADHD display elevated narcissistic traits compared to their peers [22], and these traits also predict increases in ADHD symptom severity over time [14, 27]. Thus, it is important to examine whether narcissistic traits are uniquely related to proactive and reactive functions of aggression in children at-risk for ADHD.

Therefore, the current study examined the relation between narcissism and aggression among children at-risk for ADHD. This study is the first to our knowledge to explore the relation of narcissism to specific functions of proactive and reactive aggression among children at-risk for ADHD using multiple informants regarding engagement in aggressive behaviors. Identifying youth at-risk for aggressive behaviors due to narcissistic traits could allow early intervention potentially preventing a lifelong trajectory of aggressive behaviors [30, 44, 55, 57].

## Methods

### Participants

Participants were recruited through an outpatient clinic specializing in the diagnosis and treatment of ADHD as part of a larger study. All participants invited to participate in this study were undergoing an evaluation for suspected ADHD. Exclusion criteria for the original study included being non-English speaking, outside the 7 to 13 age range, and a suspected diagnosis of autism spectrum disorder. Participants were included in the current study if either the caregiver or teacher completed the measures used to assess narcissistic traits and aggression, and the child did not meet diagnostic criteria for conduct disorder. The current sample includes 110 children (69.1% boys) ages 7–13 years (*M* = 8.52; *SD* = 1.51). Most participants were non-Hispanic White (*n* = 84; 76.4%), with remaining participants non-Hispanic Black (*n* = 22; 20%), Hispanic/Latinx (*n* = 2; 1.8%), or other race/ethnicity (*n* = 2; 1.8%). Ninety-nine children met DSM-IV criteria for ADHD, 14 met criteria for an anxiety or mood disorder, 31 met criteria for ODD, and one child met criteria for depression. Most children (89.1%) were not on psychotropic medications.

### Procedures

Caregivers were administered the Kiddie-Schedule for Affective Disorders and Schizophrenia (K-SADS; [31]) by a licensed clinical psychologist, masters-level clinician, or clinical psychology graduate student and completed ratings as part of a research study visit at a hospital. Caregivers also signed a release of information to gather teacher ratings, and the child’s teacher was invited to complete the aggression measure via mail. Both caregivers and teachers were compensated for their participation.

## Measures

### Narcissistic Traits

The Antisocial Process Screening Device (APSD; [25]) is a 20-item caregiver-report measure that assesses three dimensions of psychopathic traits: callous-unemotional (CU) traits, impulsivity, and narcissism. This measure uses a three-point scale; scores of 0 indicate the item is *not true at all*, scores of 1 indicate that the item is *sometimes true*, and scores of 2 indicate that the item is *definitely true*. Prior research has demonstrated adequate reliability for the APSD [25, 26]. However, mean internal consistency scores in the current study sample were: CU traits α = 0.39; narcissism α = 0.76; impulsivity α = 0.58. This internal consistency represents the correlation or relatedness of measure items as well as the amount of measurement error with scores of 0.70- 0.95 indicating acceptable reliability [61]. Internal consistency scores for the APSD indicate that, in the current sample, the error variance for the CU subscale and impulsivity subscale were 0.85 and 0.66, respectively. Given the significant error variance and concerns regarding internal consistency for measurement of personality [46], only scores for narcissism were used in the current study due to low reliability for both CU traits and impulsivity. The narcissism subscale includes seven items (e.g., “brags excessively about his/her abilities, accomplishments, or possessions”, “uses or ‘cons’ other people to get what he/she wants”, “can be charming at times, but in ways that seem insincere or superficial”, “thinks he/she is better or more important than other people”, and “his/her emotions seem shallow and not genuine”).

### Aggression

Caregivers and teachers completed Dodge and Coie’s [14] measure of proactive and reactive functions of aggression consisting of six items rated on a five-point scale (1 = *never*, 5 = *always*). Subscale scores for reactive aggression and proactive aggression were calculated. Adequate reliability, validity, and factor structure have been demonstrated [16, 20, 21, 39, 50, 64]. Mean internal consistency scores in the current study were: caregiver-report (reactive α = 0.82; proactive α = 0.76) and teacher-report (reactive α = 0.93; α = 0.83). Reactive aggression items include “when teased, strikes back”, “overreacts angrily to accidents”, and “blames others in fights”, whereas proactive aggression items include “uses physical force to dominate”, “gets others to gang up on a peer”, and “threatens and bullies others”.

### DSM-Based Disorders

To characterize the sample in the context of DSM-based nosology, caregivers were administered the K-SADS [31], a semi-structured diagnostic interview, to assess mental health disorders. The disruptive behavior disorder (including ADHD), mood disorder, and anxiety disorder modules were administered. If any K-SADS screening item was endorsed as positive, a full module was administered. Reliability and validity for this interview are good to excellent [31].

### ADHD and ODD Symptoms

The Vanderbilt ADHD Diagnostic Parent Rating Scale [67] was used to assess ADHD and ODD symptoms. Caregivers rated frequency of each DSM ADHD and ODD symptom on a four-point scale (0 = *never*, 3 = *very often*). Mean internal consistency scores for the ADHD and ODD scale in the current sample were α = 0.90 and α = 0.91, respectively.

## Analyses

Pearson correlations were calculated between all study variables. Multiple linear regression models examined whether narcissism was uniquely associated with proactive and reactive aggression, above and beyond ADHD symptoms, ODD symptoms, and child sex. All predictor variables and covariates in each regression model were entered simultaneously (i.e., in one regression step). Covariates were informed by the literature as ODD and ADHD are both associated with narcissism scales [56], and sex is associated with risk for aggression [35]. The aggression function (i.e., either proactive or reactive) that was not the dependent variable was also entered as a covariate (i.e., proactive aggression was included as a covariate in the model predicting reactive aggression and vice versa).

## Results

The correlation matrix is presented in Table 1. Narcissism was uniquely associated with higher ratings of caregiver-reported proactive aggression, β = 0.36, *t* (102) *=* 3.61, *p* <.01, but not teacher-report of proactive aggression. ADHD and ODD symptoms were not uniquely associated with proactive aggression. Narcissism was not associated with reactive aggression ratings by any informant. ADHD symptoms were associated with higher ratings of teacher-report of reactive aggression, β = 0.22, *t* (102) = 2.58, *p* <.05, but not caregiver-report of reactive aggression. ODD symptoms were not associated with reactive aggression. See Table 2 for complete regression results^1^.

**Table 1:** Inter-correlations and descriptive statistics

	1	2	3	4	5	6	7	8
1. APSD Narcissism	--							
2. Proactive Caregiver	0.55**	--						
3. Reactive Caregiver	0.50**	0.56**	--					
4. Proactive Teacher	0.25*	0.36**	0.37**	--				
5. Reactive Teacher	0.25**	0.29**	0.44**	0.66**	--			
6. ODD symptoms	0.63**	0.52**	0.73**	0.26**	0.26**	--		
7. ADHD symptoms	0.36**	0.35**	0.48**	0.15	0.31**	0.52**	--	
8. Sex	− 0.19*	− 0.04	− 0.02	0.07	0.13	− 0.06	− 0.04	--
Mean	3.33	1.26	2.58	1.58	2.84	1.20	1.95	n/a
*SD*	2.69	0.574	1.14	0.873	1.39	0.750	0.563	n/a
*n*	110	110	110	106	106	110	110	110

Note. APSD = Antisocial Process Screening Device; ODD = Oppositional Defiant Disorder; ADHD = Attention-Deficit/Hyperactivity Disorder; Sex was coded as 0 = Female, 1 = Male

*p <.05. **p <.01

**Table 2:** Predictors of proactive and reactive aggression among youth at-risk for ADHD

	Caregiver-Report			Teacher-Report		
	F = 14.793**			F = 17.114**		
*Proactive Aggression*	*β*	*p*	*T*	*β*	*p*	*T*
Narcissism	0.36**	< 0.01	3.61	0.06	0.567	0.57
ADHD	0.05	0.598	0.53	− 0.13	0.147	-1.46
ODD	0.01	0.921	0.10	0.11	0.279	1.09
Sex	0.04	0.632	0.48	− 0.00	0.964	− 0.05
Reactive Aggression	0.35**	0.002	3.13	0.66**	< 0.01	8.30
*Reactive Aggression*	*F* = 30.372**			*F* = 19.618**		
Narcissism	− 0.03	0.709	− 0.37	0.07	0.464	0.73
ADHD	0.11	0.124	1.55	0.22*	0.011	2.58
ODD	0.57	< 0.01	6.30	− 0.05	0.654	− 0.45
Sex	0.03	0.603	0.52	0.11	0.162	1.41
Proactive Aggression	0.25**	< 0.01	3.13	0.62**	< 0.01	8.30

Note. APSD = Antisocial Process Screening Device; ODD = Oppositional Defiant Disorder; ADHD = Attention-Deficit/Hyperactivity Disorder. * *p* <.05; ** *p* <.01; T *p* <.10

## Discussion

The current study is the first to explore the relation between proactive and reactive functions of aggression and narcissism in a sample of youth at-risk for ADHD using a multi-informant approach. This research examined narcissistic traits while controlling for other key clinical variables (e.g., ADHD, ODD, other aggression subtype), allowing for examination of the unique contribution of narcissistic traits in relation to aggression. Youth higher in narcissism were more likely to exhibit caregiver-reported proactive aggression. Narcissistic traits were not found to uniquely relate to reactive aggression. These findings are supported by previous research detailing narcissism’s role as a significant predictor of proactive aggression in a general population of children [18, 24, 37, 41, 66], and our results suggest this relation extends these findings to children at-risk for ADHD.

Higher narcissistic traits were related to increased caregiver-reported proactive aggression amongst our sample of school-age children at-risk for ADHD. Limbic and paralimbic system dysfunction characteristic of both narcissism [34] and ADHD [49, 51, 63] has also been implicated as neurological mechanisms underlying aggression in youth [68]. Additionally, proactive aggression has been defined as aggressive behavior that is anticipated to be followed by a reward [32], which may be particularly salient in the context of ADHD given children with ADHD show increased sensitivity to behaviorally-based external rewards in the context of reduced internal reward sensitivity [62]. Proactive aggression may be associated with immediate external rewards, and behavior of children with ADHD may be particularly driven by immediate reinforcement. Deficits in social information processing may also offer a potential explanation for the association between narcissism and proactive aggression. Deficits in social information processing have been related to aggression in children [32], and narcissism may be associated with cognitive schemas that influence social information processing.

Consideration of co-occurring impairments with ADHD symptoms is also important for contextualizing study results. Prior research has demonstrated that the relation between narcissism and behavioral problems in children is moderated by self-esteem, such that narcissism is more strongly related to behavioral problems in the context of low self-esteem [3]. This is an important consideration for children with symptoms of ADHD who are at greater risk for poor self-esteem, particularly for children with untreated ADHD such as those in the current sample who were not yet treated and currently undergoing evaluation for ADHD [29]. This greater risk for low self-esteem may have strengthened the association between narcissism and behavior problems such as proactive aggression in this sample. Additionally, diagnoses such as ODD or anxiety disorders are also highly comorbid with ADHD [28]. Prior research has demonstrated anxiety to be a risk factor for engagement in aggressive behavior [13], and the association between anxiety and aggression may vary across types or functions of aggression [cf. 5, 45]. Future research should further examine how co-occurring psychopathologies may influence predictors of aggression in children with ADHD. Additionally, the current study was unable to evaluate whether others psychopathic traits, including impulsivity or CU traits (or the more modern terminology of limited prosocial emotions), may moderate or influence the association between narcissism and aggression, and these associations should be examined in future research.

In contrast to findings for caregiver-reported proactive aggression, narcissism was not uniquely associated with teacher-reported proactive aggression. This could be the result of either differences or perceived differences in aggressive behavior across home and school environments. Correlates of aggressive behavior may vary across environments, and aggressive behavior may be triggered or motivated by different factors in different contexts. Interventions to address aggressive behavior may need to consider environmental context when identifying potential modifiable factors to reduce aggression. Alternatively, shared method variance (i.e., report of narcissism and proactive aggression both via caregiver-report) may also be a potential explanation for the different pattern of findings across informants. Shared variance in caregiver-reported narcissism and caregiver-reported aggression may have been due to reporting by a single reporter at a single time point resulting in an inflated estimation of the association, whereas the association between caregiver-reported narcissism and teacher-reported aggression may represent a more valid estimate of the relationship between these constructs without the bias of shared method variance. Future research should examine the relation between narcissism and objectively measured proactive aggression.

Narcissism was uniquely associated with proactive aggression but not with reactive aggression. Past research in the general population regarding the differential role of narcissism in relation to functions of aggression has been inconclusive. Some research has demonstrated narcissism may show a stronger relation to aggression when this behavior has been provoked [36], whereas other research has found narcissism may have a stronger association with proactive aggression [40, 53]. For youth with ADHD, it may be that other factors such as impairments in inhibitory control mechanisms and the impulsivity characteristic of ADHD itself are the primary predictors of reactive aggression, rather than other characteristics such as narcissism. If limited capacity for inhibition and impulsivity drive engagement in reactive aggression for youth with ADHD, reactionary aggressive behaviors may be present across youth with ADHD regardless of level of narcissism.

Findings should be interpreted within the context of study limitations. Given this was a clinic-referred sample, there may have been restriction in range in scores of aggression and narcissism, which may have limited our ability to find associations between reactive aggression and narcissistic traits. Further, measures of narcissism may have been influenced by reporter bias, and shared method variance resulting from caregiver-report of both narcissism and child aggression may have inflated estimation of relationships between those constructs. Potential bias in assessment of aggression due to reliance on caregiver- and teacher-report may also limit validity of study results. Prior research has demonstrated potential bias in teacher- and caregiver-report of child aggressive behavior due to sociodemographic factors and limited reliability between multiple reporters [1, 12, 19]. Future studies should employ behavioral observations of aggression to examine the relation of narcissistic traits with aggression among youth at-risk for ADHD. Lastly, exploration of the relation between aggression and other psychopathic traits (i.e., CU traits, impulsivity) was prevented by low reliability scores on those subscales of the APSD. The majority of score variation on those subscales was due to measurement error rather than true measurement of those constructs, meaning interpretation and generalizability of results utilizing those subscales would have been unreliable with results possibly attributable to measurement error instead of true relationships between study constructs. Future research should utilize more robust and reliable methods for assessment of CU traits and impulsivity in this population, including observational methods. For example, developed in part to overcome psychometric limitations of the APSD CU subscale, the Inventory of Callous-Unemotional Traits (ICU; [23]) is a longer and commonly used measure of CU traits which may be useful in future research in this area.

The current study identified that narcissism is a key correlate of proactive aggression among children at-risk for ADHD, and consideration of narcissistic traits may be useful in the identifying children at-risk for aggressive behaviors and poorer outcomes. By implementing interventions focusing on teaching youth to regulate narcissistic behaviors (e.g., emphasis on appropriate responses to threats, perspective taking, practicing empathy), professionals may minimize some externalizing behavior problems and possibly prevent the escalation of more severe aggression and conduct problems among youth at-risk for ADHD. Future research should investigate whether clinical interventions addressing narcissistic behaviors are effective for reducing proactive aggression among youth at-risk for ADHD. Prior research has demonstrated that parenting interventions may be an effective avenue for reducing features of psychopathy among children with externalizing problems [47]. Additionally, interventions focused on cultivating positive relationships and providing positive reinforcement may also be effective avenues for addressing psychopathy in children, and early intervention can play a key role in reduction of traits of psychopathy [8].

## Data Availability

Data is available from the study authors upon request.

## Footnotes

1 Based on a question of an anonymous reviewer, sensitivity regression analyses were conducted that included co-occurring internalizing symptoms as rated by caregivers on the Vanderbilt ADHD Diagnostic Parent Rating Scale as an additional covariate. The pattern of results was unchanged.
